# Effects of hydroxy methionine analog iron chelate on growth performance, blood parameters, and iron metabolism in weaned piglets

**DOI:** 10.1038/s41598-025-22609-8

**Published:** 2025-11-06

**Authors:** Yuemeng Fu, Jingzi Fang, Yilin Ge, Shuning Zhang, Yuhang Liu, Guohui Zhou, Xuejun Yuan, Ning Jiao, Yang Li, Weiren Yang

**Affiliations:** 1 Key Laboratory of Efficient Utilization of Non-Grain Feed Resources (Co-construction by Ministry and Province), College of Animal Science and Technology, Ministry of Agriculture and Rural Affairs, Shandong Agricultural University, Panhe Street 7, Tai’an, 271017 China; 2 Changsha Xingjia Bio-Engineeriong Co., Ltd, Changsha, 410000 Hunan China; 3 SDU-ANU Joint Science College, Shandong University, Wenhuaxilu 180, Weihai, 264209 China; 4 College of Life Sciences and Technology, Shandong Agricultural University, Daizong Street 61, Tai’an, 271018 China

**Keywords:** Chelated iron, Iron metabolism, Methionine hydroxy analog, Trace element, Weaned piglets, Animal physiology, Homeostasis

## Abstract

This study aimed to investigate the effects of dietary hydroxy methionine analog iron chelate (Fe-HMA) as an iron source on the growth performance, blood parameters, and iron metabolism of weaned piglets. A 28-day trial was conducted using 120 weaned piglets that were randomly allocated to two treatment groups with different iron sources. The Fe-sulfate group received a diet containing 100 mg Fe/kg in the form of ferrous sulfate, while the Fe-HMA group received a diet with 50 mg Fe/kg in the form of Fe-HMA. Fe-HMA, as an iron source, did not significantly affect growth performance, but improved iron apparent digestibility and tissue deposition compared with the Fe-sulfate group. Additionally, Fe-HMA significantly enhanced erythrocyte-related indicators and regulated the serum iron metabolism markers. It also upregulated the relative expression of the iron metabolism-related genes ferroportin1 (*FPN1*) in the duodenum and hepcidin antimicrobial peptide (*HAMP*), ferritin light chain (*FTL*), and transferrin (*TF*) in the liver, while downregulating transferrin receptor (*TFRC*) expression in the liver. Replacing dietary inorganic iron with HMA-chelated iron improved iron metabolism in weaned piglets, suggesting its potential as an effective alternative source of iron in porcine nutrition.

## Introduction

Iron (Fe) is an indispensable trace element that plays crucial roles in various biological activities, including serving as a critical component of hemoglobin (HGB), myoglobin, cytochromes, and iron-sulfur proteins^1,2^. Iron supplementation has been shown to improve the feed-to-gain ratio (F/G), hematological parameters, and iron metabolism indicators in piglets^3^. Insufficient iron intake can lead to adverse effects such as decreased iron deposition in tissues, iron deficiency anemia, stunted growth, and mortality in weaned piglets in severe cases^4–6^. Additionally, excessive iron supplementation may induce oxidative stress and inflammation, which can be detrimental to piglet health^4^. Ferrous sulfate is the most commonly used form of iron in commercial piglet diet. However, it is characterized by low bioavailability and high environmental excretion, potentially contributing to water and soil contamination^7,8^. Consequently, enhancing the bioavailability and utilization of iron in piglets has become an increasingly important consideration in animal nutrition research.

Organic iron, produced by microbes or combined with amino acids, peptides, and HGB through chelation or complexation, is gaining recognition as a viable alternative to inorganic iron in animal production^9–11^. Previous studies have demonstrated the advantages of organic iron over inorganic forms of iron. For instance, partially (50%) or completely (100%) replacing inorganic iron supplementation with iron-peptide complexes in the diet has been shown to increase iron deposition in the liver, kidneys, and spleen of weaned piglets^10^. Moreover, compared to dietary ferrous sulfate (104 mg/kg), dietary organic iron from yeast (84 mg/kg) exhibited higher iron apparent digestibility (AD), decreased iron excretion in the environment, and increased serum iron (SI) in weaned piglets^8^. In broilers, supplementation with 10 mg/kg iron from Fe-glycine chelate increased SI, HGB level, and red blood cell count (RBC) compared to 40 mg/kg iron sulfate supplementation^12^. These studies suggest that organic iron has higher biological availability and can reduce fecal iron excretion compared to ferrous sulfate.

Hydroxy methionine analog (HMA) chelated microminerals have been reported to improve average daily gain (ADG), immune function, antioxidant capacity, and fat thickness in pigs^13,14^. A previous study demonstrated that dietary supplementation with 50 mg Mn/kg HMA-chelated manganese improved growth performance and regulated Mn metabolism-related gene expression^15^. Additionally, dietary supplementation with 75 mg Mn/kg of HMA-chelated manganese increased liver and tibia iron deposition compared to 100 mg Mn/kg of Mn sulfate in broilers^15^. Despite these promising findings, the application of iron chelated hydroxy methionine analog (Fe-HMA) in piglets has not been reported.

Therefore, this study aimed to evaluate the effects of dietary supplementation with 50 mg Fe/kg in the form of Fe-HMA on growth performance, blood parameters, and iron metabolism in weaned piglets. The findings of this study provide a basis for the potential application of Fe-HMA in piglet nutrition and contribute to ongoing efforts to improve iron utilization in animal production.

## Materials and methods

### Animals and experimental design

A total of 120 healthy 35-d-old weaned piglets (Duroc × Landrace × Large White) with similar body weights (11.09 ± 0.16 kg) were randomly assigned to two groups (four replicates/group, 15 pigs/replicate). Piglets in the Fe-sulfate group were fed a basal diet supplemented with 100 mg Fe/kg ferrous sulfate monohydrate, as recommended by National Research Council (NRC)^16^. Those in the Fe-HMA group received a basal diet supplemented with 50 mg Fe/kg HMA-chelated iron (provided by Changsha Xingjia Bio-Engineeriong Co., Ltd., Changsha, China). The basal diet (Table 1) was formulated according to NRC^16^ recommendations to meet or exceed the nutritional requirements for weaned piglets, except for iron. The pigs were provided by Huanshan Group Co., Ltd. All piglets were intramuscularly injected with 1 mL dextran iron solution containing 100 mg iron on day 3 of age. The formal experiment began at 38 days of age and lasted for 28 d. Prior to the trial, piglets were fed a basal diet and housed in a temperature-controlled room (25–28 °C) with one replicate per pen for a three-day acclimation period. During this pre-experiment phase, the feed for piglets were gradually transitioned to the experimental diet, with a gradual shift from a 1:2 to a 2:1 ratio of ordinary feed to the feed containing different iron sources. Throughout the trial, the piglets had *ad libitum* access to water and feed containing different iron sources.

**Table 1:** Ingredient composition and nutritional values of basal diets (air-dried basis).

Items	Content
Ingredients	
Corn	59.15
Soybean meal	16.0
Expanded soybean	13.0
Fish meal	2.80
Whey	3.00
Soybean oil	2.00
Limestone	0.80
Calcium hydrogen phosphate	1.00
NaCl	0.25
Premix^†^	2.00
Total	100.0
Nutrient^‡^	
Digestible energy, MJ/kg	14.85
Crude protein, %	18.88
Standard total gastrointestinal digestibility phosphorus, %	0.36
Calcium, %	0.73
Lysine, %	1.23
Threonine, %	0.74
Methionine, %	0.47
Tryptophan, %	0.20
Methionine + Cysteine, %	0.69
Fe, mg/kg	65.88

^†^Premix composition per kg of diet: vitamin A (1750 IU), vitamin D_3_ (200 IU), vitamin E (11 IU), vitamin K_3_ (0.5 mg), vitamin B_1_ (1 mg), vitamin B_2_ (3 mg), vitamin B_12_ (15 µg), biotin (0.05 mg), folic acid (0.3 mg), pantothenic acid (9 mg), pyridoxine (3.0 mg), Mn (50 mg), Zn (95 mg), Cu (125 mg), I (0.14 mg), and Se (0.25 mg).

^‡^Nutrient levels are calculated. Iron level was determined experimentally.

### Sample collection and preparation

On day 28 of the experiment, the piglets were weighed at 07:00 h to calculate growth performance. One piglet from each replicate was selected for jugular vein blood collection. Two milliliters of blood were collected into vacuum anticoagulation tubes for immediate routine blood analysis. The remaining 10 mL blood sample were centrifuged at 3500 rpm for 15 min to obtain serum, which was then immediately stored at − 20 ℃ for iron metabolism indicator analysis. Subsequently, piglets from each replicate were euthanized by intravenous sodium pentobarbital injection, followed by exsanguination. Then the thoracic cavity was dissected. Approximately 100 g of each *longissimus dorsi* (LD), heart, liver, spleen, and kidney were obtained and stored at − 20 ℃ for trace element determination. Additionally, approximately 2 g each of the liver and duodenum were stored at − 80 ℃ to determine the relative mRNA expression of genes related to iron metabolism.

### Routine blood index analysis

RBC count, HGB content, hematocrit (HCT), mean corpuscular volume (MCV), mean corpuscular hemoglobin (MCH), mean corpuscular hemoglobin concentration (MCHC), coefficient of variation of RBC volume distribution width (RDW), standard deviation of RBC distribution width (RDW-SD), white blood cell count (WBC), lymphocyte count (Lym), monocyte count (Mon), eosinophil count (Eos), and basophil count (Bas) in whole blood were determined using a KX-21 hemocyte analyzer (SYSMEX Corporation, Kobe, Japan).

### Trace element determination

#### Trace elements in feed, feces, and tissues

Fe, Cu, Zn, and Mn contents in the feed, feces, and tissue (LD, heart, liver, kidney, and spleen) samples were determined according to the China National Standard (CNS, GB/T 13885). Briefly, the samples were pulverized, ashed at 550 ℃ for 3 h, and re-dissolved in 10 mL of hydrochloric acid (6 mol/L). The solutions were diluted to 50 mL using distilled water. The absorbance of the sample hydrochloric acid solution and standard solution was determined using a TAS-990 spectrophotometer (Beijing Puxi General Instrument Co., Ltd., Beijing, China). The wavelengths for Fe, Cu, Zn, and Mn measurements were 248.3, 324.8, 213.8, and 279.5 nm, respectively.

#### Apparent bioavailability of trace elements

Acid insoluble ash (AIA) was used as endogenous indicators according to CNS (GB/T23742-2009) (15). Briefly, polyester mesh ash-free bags containing the feed and feces samples were kept at a slight boil in 3 mol/L hydrochloric acid for half an hour. After washed and dried, the bags were ashed at 550 ℃ to calculate the AIA content in the feed and feces. The AD of micronutrients was calculated as follows: [trace element AD (%) = 1 – (bc/ad) × 100]. a, trace element content in diet (mg/kg); b, trace element content in digesta (mg/kg); c, diet AIA (g/kg); d, digesta AIA (g/kg).

### Iron metabolism indicators

Ceruloplasmin (Cp), SI, and total iron binding capacity (TIBC) in serum were determined using commercial kits from Nanjing Jiancheng Bioengineering Institute (Jiangsu, China), according to a previous study (Kwiecień et al., 2015). Ferritin (FE), transferrin (TF), soluble transferrin receptor (sTfR), and hepcidin antimicrobial peptide (HAMP) in serum were measured using ELISA kits from Jiangsu Meimian Industrial Co., Ltd. (Jiangsu, China) as reported in a previous study^9^.

### Relative mRNA expression of iron metabolism-related genes

Total RNA was extracted from the liver [hepcidin antimicrobial peptide (*HAMP*), ferritin light chain (*FTL*), ferritin heavy chain 1 (*FTH1*), transferrin (*TF*), transferrin receptor (*TFRC*), and transferrin receptor 2 (*TFR2*)] and duodenum [duodenal cytochrome b (*Dcytb*), divalent metal transporter 1 (*DMT1*), ferroportin1 (*FPN1*), *FTL*, and *FTH1*] using the AIPzol reagent (I-presci Scientific, Beijing, China) according to the manufacturer’s instructions. RNA was reverse-transcribed into cDNA using an Evo M-MLV Reverse Transcription Premix Kit (Accurate Biology, Hunan, China). All primer sequences (Table 2) were designed based on pig genes in accordance with previous research^10^. Real-time quantitative fluorescence PCR (qPCR) was conducted according to the SYBR Green Pro Taq HS premix qPCR kit protocol (Accurate Biology, Hunan, China). The relative mRNA expression of iron metabolism-related genes was calculated using the 2^−ΔΔCt^ method after normalization with β-actin as the internal reference gene.

**Table 2:** Primer sequences for quantitative real-time PCR.

Genes^†^	GenBank	Primer sequence (5’ − 3’)^‡^	Size, bp
*β-Actin*	NM_001170517.2	F: CCACGAAACTACCTTCAACTC R: TGATCTCCTTCTGCATCCTGT	131
*Dcytb1*	AM268434.1	F: CGTCGACAAAGCAACCCTCAC R: GTTGGATCCGGTTTCGTGCAG	112
*DMT1*	NM_001128440.1	F: ATCGCCATCATCCCCACTCTG R: ACTGGCCGCAAGCTTGTAAAC	146
*FPN1*	XM_013984335.2	F: TCATCATGGTCATCTTGGCTCC R: GTGACCCATTGCCACAAAGGA	83
*HAMP*	NM_214117.1	F: CGTTCTCCCATCCCAGACAAG R: CCACAGATTGCTTTGCGAGAG	159
*TF*	NM_001244653.1	F: AGTCTCTGTGCTCTGTGTATCG R: TGTTCTGCTGGACAACCTGAT	157
*FTL*	NM_001244131.1	F: CCCAGGTTCGTCAGAATTATTCCA R: CGGTTGAAATAGAAGCCCAGAGA	112
*FTH1*	NM_213975.1	F: GACTGGGAGAATGGGCTGACT R: CTTTGATGGCTTTCACCTGCTCAT	166
*TFRC*	NM_214001.1	F: TGATGCTGCTTTCCCTTTCCT R: CGTGCCATTCTGTTCAACTGAG	150
*TFR2*	XM_021086235.1	F: GATGATAAGTTCCACGCCAAGAC R: CTGAAGACCACCTGCTCGTAA	119

^†^*Dcytb1*, Duodenal Cytochrome b; *DMT1*, divalent metal transporter 1; *FPN1*, ferroportin1; *HAMP*, hepcidin antimicrobial peptide; *TF*, transferrin; *FTL*, ferritin light chain; *FTH1*, ferritin heavy chain 1; *TFRC*, transferrin receptor; *TFR2*, transferrin receptor 2.

^‡^F: forward primer; R: reverse primer.

### Statistical analysis

Replicates were used as the unit for assessing growth performance, whereas individual weaned piglets served as the experimental unit for other indices. Statistical data were analyzed using the Independent-Samples T-test, with *P* < 0.05, considered statistically significant, and 0.05 < *P* < 0.10 as a tendency of difference.

## Results

### Growth performance

The growth performance of the piglets is shown in Table 3. No significant differences were observed in the final body weight, ADG, or average daily feed intake (ADFI) between the Fe-HMA and Fe-sulfate groups (*P* > 0.05). However, the F/G ratio in the Fe-HMA group tended to decrease compared to that in the Fe-sulfate group (*P* < 0.10).

**Table 3:** Effects of replacing inorganic iron with hydroxy methionine analogue chelated iron (Fe-HMA) on production performance of piglets.

Items	Fe-sulfate^†^	Fe-HMA^‡^	SEM	*P* value
Initial body weight, kg	10.97	11.21	0.164	0.536
Final body weight, kg	26.19	26.81	0.388	0.481
ADG, kg/d	0.54	0.56	0.009	0.501
ADFI, kg/d	0.83	0.82	0.008	0.589
F/G	1.52	1.46	0.017	0.090

ADG, average daily gain; ADFI, average daily feed intake; F/G, feed-to-gain ratio; SEM, standard error of the means.

^†^The group of piglets fed a basal diet with 100 mg Fe/kg in the form of ferrous sulfate.

^‡^The group of piglets fed a basal diet with 50 mg Fe/kg in the form of Fe-HMA.

### Routine blood indices

Table 4 displays the routine blood indices. Compared with the Fe-sulfate group, dietary Fe-HMA significantly increased the RBC, HGB, MCV, MCH, RDW, RDW-SD, and Mon values (*P* < 0.05). Additionally, the HCT value tended to increase in the Fe-HMA group (*P* < 0.10). However, MCHC, WBC, Lym, Eos, and Bas values were not significantly affected by the iron source (*P* > 0.05).

**Table 4:** Effects of replacing inorganic iron with Fe-HMA on blood cell parameters in piglets.

Items	Fe-sulfate^†^	Fe-HMA^‡^	SEM	*P* value
Erythrocyte				
RBC, 10^12^/L	5.90	6.43	0.127	0.020
HGB, g/L	91.67	103.75	2.776	0.012
HCT, %	32.45	35.78	0.923	0.063
MCV, fL	50.67	53.57	0.721	0.028
MCH, pg	14.77	15.67	0.223	0.028
MCHC, g/L	290.7	289.75	1.386	0.747
RDW, %	18.90	20.27	0.181	< 0.001
RDW-SD, fL	35.20	39.23	0.781	< 0.001
Immune cells				
WBC, 10^9^/L	26.10	27.68	0.920	0.453
Lym, 10^9^/L	12.98	14.72	0.792	0.322
Mon, 10^9^/L	5.58	11.68	1.425	0.003
Eos, 10^9^/L	0.27	0.26	0.032	0.964
Bas, 10^9^/L	0.12	0.15	0.018	0.570

RBC, red blood cell count; HGB, hemoglobin content; HCT, hematocrit; MCV, mean corpuscular volume; MCH, mean corpuscular hemoglobin; MCHC, mean corpuscular hemoglobin concentration; RDW, coefficient of variation of erythrocyte distribution width; RDW-SD, standard deviation of red blood cell distribution width; WBC, white blood cell count; Lym, Lymphocyte count; Mon, Monocyte count; Eos, eosinophil count; Bas, basophil count; SEM, standard error of the means.

^†^The group of piglets fed a basal diet with 100 mg Fe/kg in the form of ferrous sulfate.

^‡^The group of piglets fed a basal diet with 50 mg Fe/kg in the form of Fe-HMA.

### AD and excretion of trace elements

As shown in Table 5, Fe-HMA significantly increased iron AD and decreased iron excretion compared with the Fe-sulfate group (*P* < 0.05). However, no significant differences were observed in the AD and excretion of Zn, Cu, or Mn between the two groups (*P* > 0.05).

**Table 5:** Effects of replacing inorganic iron with Fe-HMA on the apparent availability and excretion of iron, copper, zinc, and manganese in piglets.

Items	Fe-sulfate^†^	Fe-HMA^‡^	SEM	*P* value
Apparent availability, %				
Fe	34.92	41.51	1.513	0.001
Cu	26.88	30.39	1.378	0.238
Zn	38.27	37.14	1.993	0.811
Mn	36.49	31.14	3.172	0.461
Excretion, mg/kg				
Fe	2046.49	1741.18	77.601	0.021
Cu	720.96	805.52	44.827	0.405
Zn	1656.66	1745.04	31.677	0.186
Mn	794.56	760.67	25.000	0.559

SEM, standard error of the means.

^†^The group of piglets fed a basal diet with 100 mg Fe/kg in the form of ferrous sulfate.

^‡^The group of piglets fed a basal diet with 50 mg Fe/kg in the form of Fe-HMA.

### Trace element deposition in tissues

Table 6 presents the trace element deposition in the various tissues. Fe-HMA significantly elevated the iron concentration in the LD, liver, and spleen (*P* < 0.05) compared to the Fe-sulfate group. Additionally, there was a trend towards increased iron deposition in the heart in the Fe-HMA group (*P* < 0.10). Cu deposition was lower in the kidney but higher in the spleens of piglets in the Fe-HMA group (*P* < 0.05). Zn deposition was significantly increased in the heart but decreased in the liver and spleen in the Fe-HMA group compared to that in the Fe-sulfate group (*P <* 0.05). However, liver Mn levels were significantly lower (*P <* 0.05), and kidney Mn levels tended to be lower (*P* < 0.10) in the Fe-HMA group than in the Fe-sulfate group. No significant differences were observed between the two groups in Fe levels in the kidney; Cu levels in the LD, heart, and liver; Zn levels in the LD and kidney; and Mn levels in the LD and spleen (*P* > 0.05).

**Table 6:** Effects of replacing inorganic iron with Fe-HMA on iron, copper, zinc and manganese deposition in tissues of piglets.

Items, mg/kg	Fe-sulfate^†^	Fe-HMA^‡^	SEM	*P* value
LD				
Fe	5.89	7.34	0.357	0.011
Cu	0.41	0.46	0.022	0.307
Zn	11.83	11.45	0.436	0.712
Mn	0.18	0.19	0.021	0.760
Heart				
Fe	23.51	26.82	0.997	0.091
Cu	1.87	2.12	0.084	0.135
Zn	11.90	15.03	0.853	0.045
Mn	0.29	0.27	0.017	0.592
Liver				
Fe	26.36	74.71	10.98	< 0.001
Cu	12.75	11.10	0.596	0.189
Zn	814.88	690.19	28.73	0.001
Mn	3.02	2.77	0.064	0.024
Kidney				
Fe	23.55	26.78	1.274	0.242
Cu	66.62	51.40	4.298	0.060
Zn	76.55	75.38	2.133	0.816
Mn	1.60	1.35	0.072	0.073
Spleen				
Fe	77.57	127.73	11.399	< 0.001
Cu	0.72	0.78	0.017	0.031
Zn	20.47	18.98	0.358	0.008
Mn	0.31	0.27	0.014	0.288

LD, *longissimus dorsi*; SEM, standard error of the means.

^†^The group of piglets fed a basal diet with 100 mg Fe/kg in the form of ferrous sulfate.

^‡^The group of piglets fed a basal diet with 50 mg Fe/kg in the form of Fe-HMA.

### Indicators of iron metabolism in serum

Table 7 displays the levels of indicators of iron metabolism. The concentrations of SI, FE, and TF in the serum were significantly higher in the Fe-HMA group (*P* < 0.05). Conversely, Cp activity and the levels of sTfR and TIBC in the serum were significantly decreased (*P* < 0.05). No significant differences were observed in the serum HAMP concentrations (*P* > 0.05).

**Table 7:** Effects of replacing inorganic iron with hydroxy methionine analogue chelated iron on iron metabolism indicators in serum of piglets.

Items	Fe-sulfate^†^	Fe-HMA^‡^	SEM	*P* value
Serum				
Cp, U/L	111.84	92.34	4.591	0.017
SI, µmol/L	19.60	34.82	3.423	< 0.001
TIBC, µmol/L	83.65	56.15	6.431	0.003
FE, ng/mL	60.24	125.16	14.712	< 0.001
TF, g/L	4.68	8.17	0.797	< 0.001
sTfR, nmol/L	62.28	42.85	4.765	0.011
HAMP, ng/mL	88.10	99.12	4.235	0.226

Cp, ceruloplasmin; SI, serum iron; TIBC, total iron binding capacity; FE, ferritin; TF, transferrin; sTfR, soluble transferrin receptor; HAMP: hepcidin antimicrobial peptide; SEM, standard error of the means.

^†^The group of piglets fed a basal diet with 100 mg Fe/kg in the form of ferrous sulfate.

^‡^The group of piglets fed a basal diet with 50 mg Fe/kg in the form of hydroxy methionine analogue iron chelate.

### Relative mRNA expression of iron metabolism-related genes in the duodenum

**Fig. 1:**
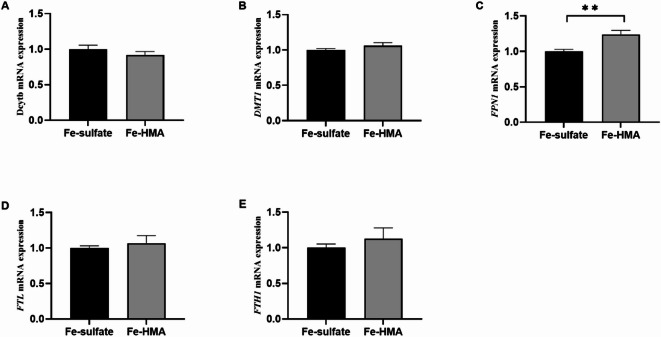
Effect of replacing inorganic iron with hydroxy methionine analogue chelated iron (Fe-HMA) on relative expression of duodenal genes in piglets. (**A**) *Dcytb* Duodenal Cytochrome b; (**B**) *DMT1* divalent metal transporter 1; (**C**) *FPN1* ferroportin1; (**D**) *FTL* ferritin light chain; (**E**) *FTH1* ferritin heavy chain 1. Fe-sulfate, piglets fed the basal diet with 100 mg Fe/kg in the forms of ferrous sulfate monohydrate; Fe-HMA, piglets fed the basal diet with 50 mg Fe/kg in the forms of Fe-HMA. Values are depicted as means ± SEM (standard error of means). **P < 0.01.

Figure 1 shows the mRNA expression levels of iron metabolism-related genes in the duodenum. The Fe-HMA group showed a significant increase in the relative mRNA expression of *FPN1* compared with that in the Fe-sulfate group (*P* < 0.05). However, no significant differences were observed in the relative mRNA expression of *Dcytb*, *DMT1*, *FTL*, or *FTH1* between the two groups (*P* > 0.05).

### Relative mRNA expression of iron metabolism-related genes in the liver

**Fig. 2:**
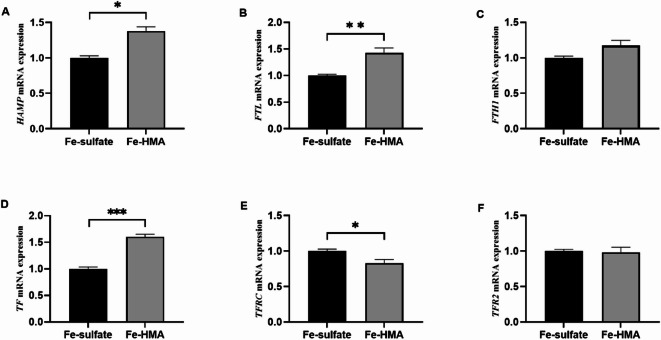
Effects of replacing inorganic iron with Fe-HMA on relative expression of liver genes in piglets. (**A**) *HAMP* hepcidin antimicrobial peptide; (**B**) *FTL*; (**C**) *FTH1*; (**D**) *TF* transferrin; (**E**) *TFRC* transferrin receptor; (**F**) *TFR2* transferrin receptor 2. Fe-sulfate, piglets fed the basal diet with 100 mg Fe/kg in the forms of ferrous sulfate monohydrate; Fe-HMA, piglets fed the basal diet with 50 mg Fe/kg in the forms of Fe-HMA. Values are depicted as means ± SEM (standard error of means). * *P* < 0.05, **P < 0.01, ***P < 0.001.

The mRNA expression levels of iron metabolism-related genes in the liver are shown in Fig. 2. Dietary Fe-HMA significantly increased the relative mRNA expressions of *HAMP*, *FTL*, and *TF* in the liver compared to that in the Fe-sulfate group, but significantly decreased that of *TFRC* (*P* < 0.05). No statistically significant differences were detected in the relative mRNA expression of *FTH1* or *TFR2* between the two groups (*P* > 0.05).

## Discussion

In the present study, replacing 100 mg/kg ferrous sulfate with 50 mg Fe/kg Fe-HMA had no adverse effects on the growth performance of weaned piglets. This finding aligns with previous research demonstrating that lower doses of organic iron sources can maintain a growth performance comparable to that of higher doses of inorganic iron. For instance, dietary supplementation with 30 and 60 mg/kg iron glycine chelate showed growth performance similar to that of 120 mg/kg ferrous sulfate heptahydrate^17^. Similarly, 50 and 100 mg/kg iron carbo-amino-phospho-chelate as an iron source did not negatively affect ADG or F/G compared with 150 mg/kg iron sulfate supplementation in weanling piglets^18^. Iron deficiency impairs the growth of piglets^19^. The results of this study suggest that a lower dose of Fe-HMA did not trigger iron metabolism disturbances or insufficient iron storage, indicating that Fe-HMA could replace Fe-sulfate as the dietary iron source.

Erythrocyte-related indicators reflect the body’s iron status and serve as crucial markers of iron absorption and utilization efficiency. In this study, Fe-HMA supplementation significantly increased RBC, HGB, HCT, MCV, and MCH in weaned piglets compared with the inorganic iron group. These parameters play vital roles in various physiological processes: RBCs participate in glycolysis, the pentose phosphate pathway, and the transport of oxygen and nutrients^20^. Hemoglobin, which contains iron, is responsible for oxygen transport^21^. The formation of RBCs requires iron, and iron deficiency anemia leads to decreased RBC, HGB, HCT, and MCV^22–24^. HGB levels below 100 g/L are considered indicative of iron deficiency in pigs^25^. Previous studies have shown that both high-dose dietary inorganic iron (3000 mg/kg) and organic iron complexes can increase these erythrocyte-related parameters^26,27^. Compared with ferrous sulfate as the iron source, feeding sows Fe-Gly significantly increased RBC counts in neonatal pigs on day 1 and HGB on day 21^28^. Therefore, the elevated levels of RBC, HCT, MCH, and HGB observed in the present study suggest better absorption and utilization of iron in piglets. However, the significantly increased RDW in Fe-HMA warrants further investigation, as it may be associated with inflammation^29^. Li et al.^4^ reported that high-iron supplementation upregulated inflammatory cytokine genes in the intestine. An increased Mon count in serum immune cells indicates potential inflammation^30^. These findings highlight the need for further research to elucidate the relationship between Fe-HMA supplementation and inflammatory response.

The AD of microminerals reflects the extent to which they are absorbed and utilized. Dietary levels of organic iron produced by *Saccharomyces cerevisiae* significantly increased the AD of iron compared with ferrous sulfate in weaned piglets^8^. In the present study, dietary Fe-HMA as the iron source similarly increased iron AD compared with ferrous sulfate. This improved digestibility likely contributed to the enhanced iron utilization observed in this study. Iron deposition in tissues serves as an indicator of iron status in animals^15^. A previous study demonstrated that supplementation of 100 mg Fe/kg in the form of heme significantly increased iron deposition in the liver, spleen, and LD muscle of weaned piglets compared to the ferrous sulfate supplementation group^9^. Consistent with these findings, dietary Fe-HMA increased iron deposition in the liver, heart, spleen, and LD muscles of weaned pigs in the present study. Although Fe-HMA enhanced iron uptake and deposition in tissues, excessive iron accumulation may induce toxicity, potentially through mechanisms such as the Fenton reaction^31^. The duration of such iron-induced toxicity remains uncertain, and its potential impact on subsequent growth and fattening is yet to be determined. Further research is needed to investigate and clarify these associated risks. There is antagonism among trace elements such as iron, copper, zinc, and manganese^6^. Interestingly, while Fe-HMA supplementation did not affect the AD and excretion levels of other elements, it did alter the deposition levels of certain elements in different organs. This observation is in consistent with a previous study that reported decreased iron excretion with supplementation of 100 mg/kg Fe glycine chelate^3^. Further research is needed to determine whether these differences are attributable to improved iron utilization or lower levels of iron supplementation in the present study. Overall, Fe-HMA improved the iron absorption and deposition.

SI, TIBC, FE, TF, and HAMP are critical markers for evaluating and tracking iron deficiency and overload^32,33^. SI mainly originates from dietary iron absorbed in the duodenum, as well as iron recycled from senescent RBCs and other tissues phagocytosed by macrophages^28,32^. FE is the main storage form of iron in vivo, reflecting the iron reserves^3,34^. Divalent iron ions in blood circulation can be oxidized into trivalent iron ions by Cp and then chelated with TF to be transported to tissues and cells. Next, iron ions combined with sTfR are transported into the cells through endocytosis^35^. Cp expression is upregulated during iron deficiency to maintain iron homeostasis^36^. Chen et al.^37^ found that dietary 800 mg/kg ferrous sulfate monohydrate significantly increased SI in piglets. Zhuo et al.^9^ supplemented weanling piglets’ diets with 100 mg Fe/kg from two iron sources, iron glycine chelate and heme, both of which significantly increased the SI level compared to the inorganic iron group. Zeng et al.^38^ supplemented 120 mg/kg iron-rich *Candida utilis* preparation and iron-rich *Lactobacillus plantarum* preparation to the diet as iron sources of weaned piglets and demonstrated that both iron source improved the absorption of iron in pigs, thus increasing the SI, TIBC, FE, and Cp levels in serum. TIBC was decreased by iron dextran treatment in neonatal pigs^39^. In this study, elevated SI, FE, and TF levels and decreased Cp, TIBC, and sTfR levels indicated the more effective absorption and better iron status compared with ferrous sulfate supplementation in pigs. HAMP, a peptide secreted by the liver, plays a crucial role in regulating iron homeostasis by inhibiting intestinal iron absorption and release from macrophages and the liver^40^. Iron overload triggers increased plasma HAMP levels in patients with DIOS^41^. High-dose administration of 9.6 mg/(100 g body weight) iron dextran to mice significantly increased the protein levels of HAMP and FE, while decreasing the levels of TFRC (TfR1) in the liver of mice^42^. However, while Fe-HMA influenced other iron metabolism indicators, no significant increase in HAMP levels was observed. This may suggest that Fe-HMA did not lead to excess iron accumulation in the body, but instead provided more bioavailable iron compared to inorganic iron. Collectively, the addition of Fe-HMA increased the absorption and utilization of iron to maintain iron homeostasis in weaned piglets.

We further investigated the mRNA expression of iron metabolism-related genes. Iron ions from food are transported to the cytoplasm of intestinal epithelial cells by DMT1, which is located at the duodenal brush border^43^. Previous studies have shown that iron deficiency can upregulate the relative expression of *DMT1* in the duodenum^44^. In the present study, no significant change was observed in the relative mRNA expression of *DMT1* in the duodenum, suggesting that iron deficiency may not be present when compared to ferrous sulfate supplementation. FPN1 is responsible for transferring iron to the circulation in vivo and is the first and only known protein that exports iron from the cytoplasm in mammals^45^. Unlike the downregulation of *FPN1* expression induced by adding iron glycine chelate to the diet of iron-deficient anemic piglets to elevate iron stores^46^, Fe-HMA significantly upregulated the relative expression of *FPN1*. Gao et al.^47^ observed increased relative expression of *FPN1* to meet high iron requirements in rats during the late pregnancy and early lactation periods. Therefore, dietary Fe-HMA may promote the utilization of absorbed iron by enhancing the function of FPN1, thereby increasing the transport of iron from cells to the blood circulation. The liver is the primary organ involved in iron metabolism. Iron ions carried by TF are transported to cells via TFRC^45^. FTL is an important form of iron storage in tissues^44^. Study has shown that a high dose of 1250 mg Fe/kg significantly increased *HAMP*, *FPN*, and *FTL* mRNA expression in mice^31^. Dietary supplementation with 797 mg Fe/kg significantly increased the relative expression of *HAMP* and reduced the relative expression of *TFRC* in weaned pigs^48^. Supplementation of sows with ferrous N-carbamylglycinate chelation at 80 mg Fe/kg significantly increased the gene expression of *TF* and decreased the mRNA expression of *TfR1* in the liver of newborn piglets^49^. In the present study, we observed upregulated *TF* and *FTL* relative expression, along with downregulation of *TFRC* relative expression in the liver, suggesting that Fe-HMA may improve iron transport and storage capacity. The upregulated *HAMP* expression in the Fe-HMA group may be a response to the high iron absorption and deposition in the liver^31^. Notably, the functional roles of iron metabolism related genes expression are also influenced by subsequent processes such as transcription and translation, which require further investigation.

## Conclusions

Overall, this study evaluated the effects of Fe-HMA as an iron source on iron metabolism in weaned piglets, in comparison to conventional inorganic iron. The administration of Fe-HMA at a low dose did not impair the growth performance of the weaned piglets, while improving erythrocyte parameters, enhancing iron absorption, utilization, and deposition, and modulating intestinal and hepatic iron metabolism at the genetic level. These results indicate that Fe-HMA represents a viable alternative to traditional iron sources and can be incorporated into the diet of weaned piglets as an effective iron supplement. However, its economic benefits still require further evaluation, particularly regarding cost savings from reduced iron supplementation, pig production, feed efficiency, and manure management, to fully assess its economic value in the livestock industry.

## Data Availability

The data are available from the corresponding author upon reasonable request.
